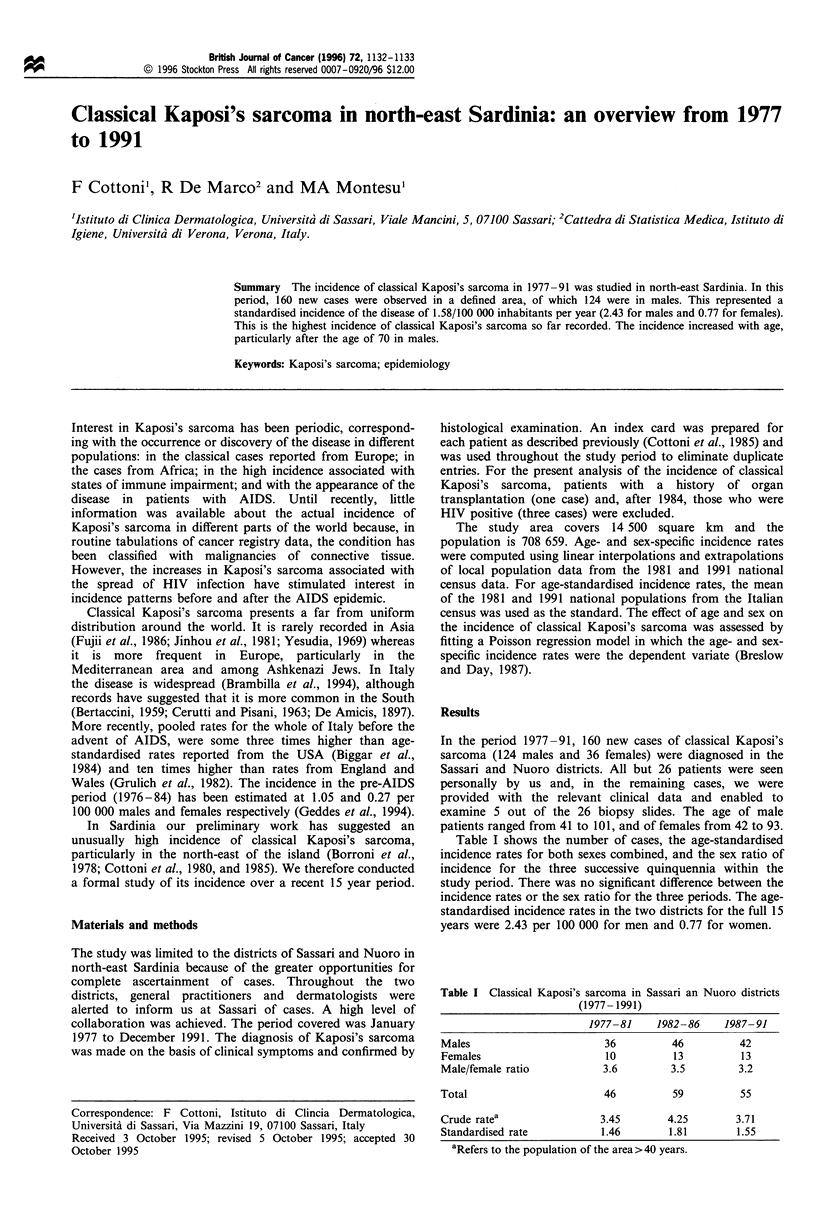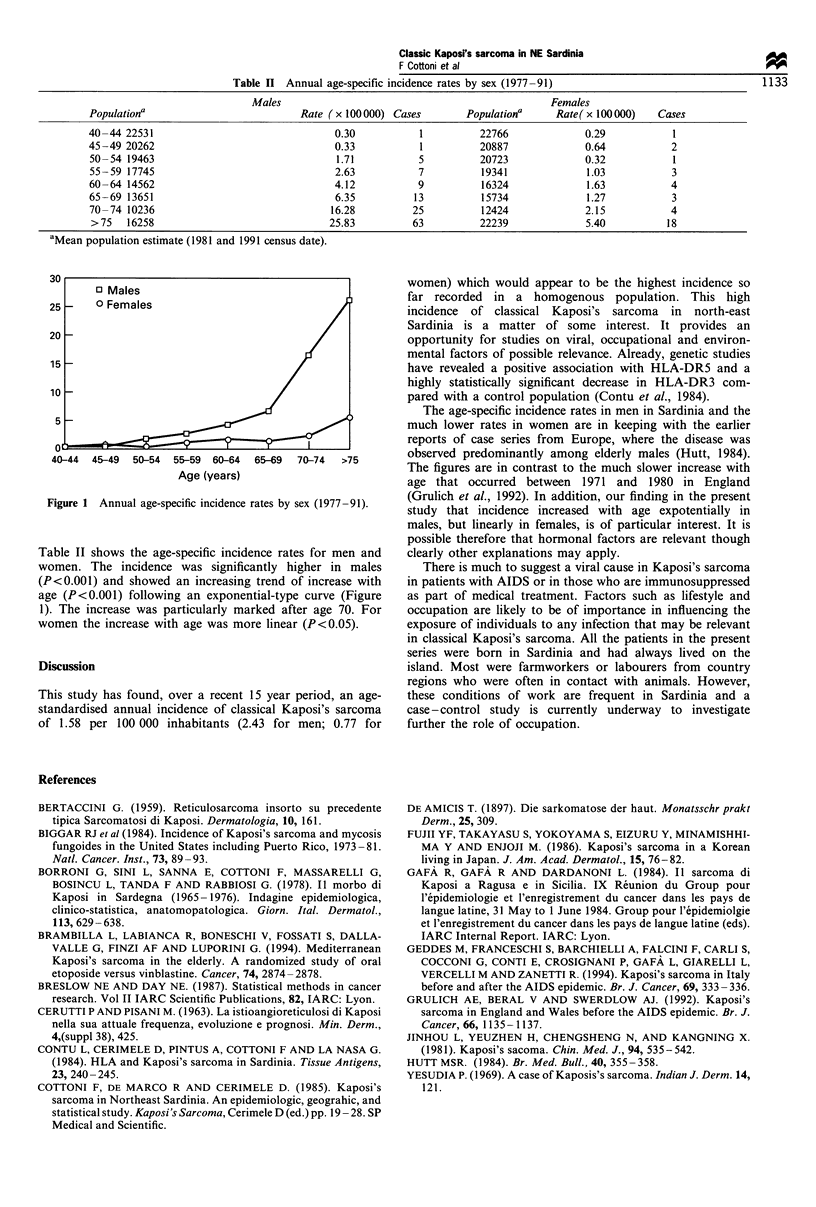# Classical Kaposi's sarcoma in north-east Sardinia: an overview from 1977 to 1991.

**DOI:** 10.1038/bjc.1996.217

**Published:** 1996-05

**Authors:** F. Cottoni, R. De Marco, M. A. Montesu

**Affiliations:** Istituto di Clinica Dermatologica, Università di Sassari, Italy.

## Abstract

The incidence of classical Kaposi's sarcoma in 1977-91 was studied in north-east Sardinia. In this period, 160 new cases were observed in a defined area, of which 124 were in males. This represented a standardised incidence of the disease of 1.58/100,000 inhabitants per year (2.43 for males and 0.77 for females). This is the highest incidence of classical Kaposi's sarcoma so far recorded. The incidence increased with age, particularly after the age of 70 in males.


British Journal of Cancer (1996) 72, 1132-1133
© 1996 Stockton Press All rights reserved 0007-0920/96 $12.00

### Classical Kaposi's sarcoma in north-east Sardinia: an overview from 1977 to 1991

F Cottoni[1], R De Marco[2] and MA Montesu[1]

[1]*Istituto di Clinica Dermatologica, Università di Sassari, Viale Mancini, 5, 07100 Sassari;* [2]*Cattedra di Statistica Medica, Istituto di Igiene, Università di Verona, Verona, Italy.*

**Summary** The incidence of classical Kaposi's sarcoma in 1977–91 was studied in north-east Sardinia. In this period, 160 new cases were observed in a defined area, of which 124 were in males. This represented a standardised incidence of the disease of 1.58/100 000 inhabitants per year (2.43 for males and 0.77 for females). This is the highest incidence of classical Kaposi's sarcoma so far recorded. The incidence increased with age, particularly after the age of 70 in males.

**Keywords:** Kaposi's sarcoma; epidemiology

Interest in Kaposi's sarcoma has been periodic, corresponding with the occurrence or discovery of the disease in different populations: in the classical cases reported from Europe; in the cases from Africa; in the high incidence associated with states of immune impairment; and with the appearance of the disease in patients with AIDS. Until recently, little information was available about the actual incidence of Kaposi's sarcoma in different parts of the world because, in routine tabulations of cancer registry data, the condition has been classified with malignancies of connective tissue. However, the increases in Kaposi's sarcoma associated with the spread of HIV infection have stimulated interest in incidence patterns before and after the AIDS epidemic.

Classical Kaposi's sarcoma presents a far from uniform distribution around the world. It is rarely recorded in Asia (Fujii *et al.*, 1986; Jinhou *et al.*, 1981; Yesudia, 1969) whereas it is more frequent in Europe, particularly in the Mediterranean area and among Ashkenazi Jews. In Italy the disease is widespread (Brambilla *et al.*, 1994), although records have suggested that it is more common in the South (Bertaccini, 1959; Cerutti and Pisani, 1963; De Amicis, 1897). More recently, pooled rates for the whole of Italy before the advent of AIDS, were some three times higher than age-standardised rates reported from the USA (Biggar *et al.*, 1984) and ten times higher than rates from England and Wales (Grulich *et al.*, 1982). The incidence in the pre-AIDS period (1976–84) has been estimated at 1.05 and 0.27 per 100 000 males and females respectively (Geddes *et al.*, 1994).

In Sardinia our preliminary work has suggested an unusually high incidence of classical Kaposi's sarcoma, particularly in the north-east of the island (Borroni *et al.*, 1978; Cottoni *et al.*, 1980, and 1985). We therefore conducted a formal study of its incidence over a recent 15 year period.

### Materials and methods

The study was limited to the districts of Sassari and Nuoro in north-east Sardinia because of the greater opportunities for complete ascertainment of cases. Throughout the two districts, general practitioners and dermatologists were alerted to inform us at Sassari of cases. A high level of collaboration was achieved. The period covered was January 1977 to December 1991. The diagnosis of Kaposi's sarcoma was made on the basis of clinical symptoms and confirmed by histological examination. An index card was prepared for each patient as described previously (Cottoni *et al.*, 1985) and was used throughout the study period to eliminate duplicate entries. For the present analysis of the incidence of classical Kaposi's sarcoma, patients with a history of organ transplantation (one case) and, after 1984, those who were HIV positive (three cases) were excluded.

The study area covers 14 500 square km and the population is 708 659. Age- and sex-specific incidence rates were computed using linear interpolations and extrapolations of local population data from the 1981 and 1991 national census data. For age-standardised incidence rates, the mean of the 1981 and 1991 national populations from the Italian census was used as the standard. The effect of age and sex on the incidence of classical Kaposi's sarcoma was assessed by fitting a Poisson regression model in which the age- and sex-specific incidence rates were the dependent variate (Breslow and Day, 1987).

### Results

In the period 1977–91, 160 new cases of classical Kaposi's sarcoma (124 males and 36 females) were diagnosed in the Sassari and Nuoro districts. All but 26 patients were seen personally by us and, in the remaining cases, we were provided with the relevant clinical data and enabled to examine 5 out of the 26 biopsy slides. The age of male patients ranged from 41 to 101, and of females from 42 to 93.

Table I shows the number of cases, the age-standardised incidence rates for both sexes combined, and the sex ratio of incidence for the three successive quinquennia within the study period. There was no significant difference between the incidence rates or the sex ratio for the three periods. The age-standardised incidence rates in the two districts for the full 15 years were 2.43 per 100 000 for men and 0.77 for women.

**Table I** Classical Kaposi's sarcoma in Sassari an Nuoro districts (1977–1991)

| | *1977–81* | *1982–86* | *1987–91* |
|---|---|---|---|
| Males | 36 | 46 | 42 |
| Females | 10 | 13 | 13 |
| Male/female ratio | 3.6 | 3.5 | 3.2 |
| Total | 46 | 59 | 55 |
| Crude rate[a] | 3.45 | 4.25 | 3.71 |
| Standardised rate | 1.46 | 1.81 | 1.55 |

[a]Refers to the population of the area > 40 years.

Correspondence: F Cottoni, Istituto di Clincia Dermatologica, Università di Sassari, Via Mazzini 19, 07100 Sassari, Italy
Received 3 October 1995; revised 5 October 1995; accepted 30 October 1995

Table II Annual age-specific incidence rates by sex (1977–91)

| | Males | | | Females | | |
|---|---|---|---|---|---|---|
| | Population[a] | Rate (×100 000) | Cases | Population[a] | Rate (×100 000) | Cases |
| 40–44 | 22531 | 0.30 | 1 | 22766 | 0.29 | 1 |
| 45–49 | 20262 | 0.33 | 1 | 20887 | 0.64 | 2 |
| 50–54 | 19463 | 1.71 | 5 | 20723 | 0.32 | 1 |
| 55–59 | 17745 | 2.63 | 7 | 19341 | 1.03 | 3 |
| 60–64 | 14562 | 4.12 | 9 | 16324 | 1.63 | 4 |
| 65–69 | 13651 | 6.35 | 13 | 15734 | 1.27 | 3 |
| 70–74 | 10236 | 16.28 | 25 | 12424 | 2.15 | 4 |
| >75 | 16258 | 25.83 | 63 | 22239 | 5.40 | 18 |

[a]Mean population estimate (1981 and 1991 census date).

**Figure 1** Annual age-specific incidence rates by sex (1977–91).

Table II shows the age-specific incidence rates for men and women. The incidence was significantly higher in males ($P<0.001$) and showed an increasing trend of increase with age ($P<0.001$) following an exponential-type curve (Figure 1). The increase was particularly marked after age 70. For women the increase with age was more linear ($P<0.05$).

### Discussion

This study has found, over a recent 15 year period, an age-standardised annual incidence of classical Kaposi's sarcoma of 1.58 per 100 000 inhabitants (2.43 for men; 0.77 for women) which would appear to be the highest incidence so far recorded in a homogenous population. This high incidence of classical Kaposi's sarcoma in north-east Sardinia is a matter of some interest. It provides an opportunity for studies on viral, occupational and environmental factors of possible relevance. Already, genetic studies have revealed a positive association with HLA-DR5 and a highly statistically significant decrease in HLA-DR3 compared with a control population (Contu *et al.*, 1984).

The age-specific incidence rates in men in Sardinia and the much lower rates in women are in keeping with the earlier reports of case series from Europe, where the disease was observed predominantly among elderly males (Hutt, 1984). The figures are in contrast to the much slower increase with age that occurred between 1971 and 1980 in England (Grulich *et al.*, 1992). In addition, our finding in the present study that incidence increased with age expotentially in males, but linearly in females, is of particular interest. It is possible therefore that hormonal factors are relevant though clearly other explanations may apply.

There is much to suggest a viral cause in Kaposi's sarcoma in patients with AIDS or in those who are immunosuppressed as part of medical treatment. Factors such as lifestyle and occupation are likely to be of importance in influencing the exposure of individuals to any infection that may be relevant in classical Kaposi's sarcoma. All the patients in the present series were born in Sardinia and had always lived on the island. Most were farmworkers or labourers from country regions who were often in contact with animals. However, these conditions of work are frequent in Sardinia and a case–control study is currently underway to investigate further the role of occupation.

### References

BERTACCINI G. (1959). Reticulosarcoma insorto su precedente tipica Sarcomatosi di Kaposi. *Dermatologia*, **10**, 161.

BIGGAR RJ *et al* (1984). Incidence of Kaposi's sarcoma and mycosis fungoides in the United States including Puerto Rico, 1973–81. *Natl. Cancer. Inst.*, **73**, 89–93.

BORRONI G, SINI L, SANNA E, COTTONI F, MASSARELLI G, BOSINCU L, TANDA F AND RABBIOSI G. (1978). Il morbo di Kaposi in Sardegna (1965–1976). Indagine epidemiologica, clinico-statistica, anatomopatologica. *Giorn. Ital. Dermatol.*, **113**, 629–638.

BRAMBILLA L, LABIANCA R, BONESCHI V, FOSSATI S, DALLA-VALLE G, FINZI AF AND LUPORINI G. (1994). Mediterranean Kaposi's sarcoma in the elderly. A randomized study of oral etoposide versus vinblastine. *Cancer*, **74**, 2874–2878.

BRESLOW NE AND DAY NE. (1987). Statistical methods in cancer research. Vol II IARC Scientific Publications, **82**, IARC: Lyon.

CERUTTI P AND PISANI M. (1963). La istioangioreticulosi di Kaposi nella sua attuale frequenza, evoluzione e prognosi. *Min. Derm.*, **4**,(suppl 38), 425.

CONTU L, CERIMELE D, PINTUS A, COTTONI F AND LA NASA G. (1984). HLA and Kaposi's sarcoma in Sardinia. *Tissue Antigens*, **23**, 240–245.

COTTONI F, DE MARCO R AND CERIMELE D. (1985). Kaposi's sarcoma in Northeast Sardinia. An epidemiologic, geograhic, and statistical study. *Kaposi's Sarcoma*, Cerimele D (ed.) pp. 19–28. SP Medical and Scientific.

DE AMICIS T. (1897). Die sarkomatose der haut. *Monatsschr prakt Derm.*, **25**, 309.

FUJII YF, TAKAYASU S, YOKOYAMA S, EIZURU Y, MINAMISHHIMA Y AND ENJOJI M. (1986). Kaposi's sarcoma in a Korean living in Japan. *J. Am. Acad. Dermatol.*, **15**, 76–82.

GAFÀ R, GAFÀ R AND DARDANONI L. (1984). Il sarcoma di Kaposi a Ragusa e in Sicilia. IX Rèunion du Group pour l'épidemiologie et l'enregistrement du cancer dans les pays de langue latine, 31 May to 1 June 1984. Group pour l'épidemiolgie et l'enregistrement du cancer dans les pays de langue latine (eds). IARC Internal Report. IARC: Lyon.

GEDDES M, FRANCESCHI S, BARCHIELLI A, FALCINI F, CARLI S, COCCONI G, CONTI E, CROSIGNANI P, GAFÀ L, GIARELLI L, VERCELLI M AND ZANETTI R. (1994). Kaposi's sarcoma in Italy before and after the AIDS epidemic. *Br. J. Cancer*, **69**, 333–336.

GRULICH AE, BERAL V AND SWERDLOW AJ. (1992). Kaposi's sarcoma in England and Wales before the AIDS epidemic. *Br. J. Cancer*, **66**, 1135–1137.

JINHOU L, YEUZHEN H, CHENGSHENG N, AND KANGNING X. (1981). Kaposi's sacoma. *Chin. Med. J.*, **94**, 535–542.

HUTT MSR. (1984). *Br. Med. Bull.*, **40**, 355–358.

YESUDIA P. (1969). A case of Kaposis's sarcoma. *Indian J. Derm.* **14**, 121.